# Enhanced wound healing by topical administration of d-limonene in alloxan induced diabetic mice through reduction of pro-inflammatory markers and chemokine expression

**DOI:** 10.1186/1471-2164-15-S2-P29

**Published:** 2014-04-02

**Authors:** Majid Ahmad, Tajdar H Khan, Mohd N Ansari, Sheikh F Ahmad

**Affiliations:** 1 Department of Pharmacology, College of Pharmacy, Salman Bin Abdulaziz University, P.O. Box 173, Alkharj 11942, Saudi Arabia; 2 Department of Pharmacology and Toxicology, College of Pharmacy, King Saud University, PO. Box 11451, Riyadh, Saudi Arabia

## Background

Delayed wound healing constitutes one of the most serious diabetes-associated complications. Diabetes can hinder the normal wound healing process by inducing long-term inflammation which can lead to delayed maturation of granulation tissues, reduced wound parallel tension and inhibition of angiogenesis [1]. D-limonene, one of the major constituent in citrus essential oils is considered to have antioxidant, hypoglycemic and anti-inflammatory activities [2].

## Materials and methods

In the present study, we investigated the effects of topical administration of d-limonene (50mg/kg and 100mg/kg; daily in acetone) on the key mediators of wound healing, namely T cell subsets, glucocorticoid-induced tumour necrosis factor receptor (GITR) expressing cells, CD4+CD25+Foxp3+ regulatory T (Treg) cells, Th17 cells, Th1 cytokines, and inflammatory mediator gene expression. In this study, five groups of Swiss albino mice were used (10 mice per group): group 1, the non-diabetic (normal control; N+M); group 2, wound in non-diabetic mice (N+W); group 3, wound in diabetic mice (D+W); group 4, Limonene 50mg/kg treated wounds in diabetic mice (D+W+L50); group 5, Limonene 100mg/kg treated wounds in diabetic mice. Wound size was recorded on every third day and after 14 days of treatment, the heparinised whole blood and the wound tissue of all the groups was collected and tested.

## Results

Limonene treated mice showed a significant decrease in wound size, the levels of GITR-expressing cells, and Th1 cytokines as well as substantial down regulation of mRNA expression of the inflammatory mediators compared with the vehicle-treated and diabetic mice. Limonene also significantly up regulated the number of Tregs or it also induced Th17/Treg balance and modulated various pro-inflammatory and anti-inflammatory cytokines and the gene expression of their mediators that mediate inflammation. This might contribute to its enhanced wound healing. Furthermore, histopathological examination showed complete re-epithelisation, decreased inflammatory cells and presence of granulation tissue in the limonene treated mice.

## Conclusions

These characteristics suggest a beneficial role for d-limonene in rebalancing the wound environment in diabetes and therefore promote healing.

## References

[B1] Daniel MC Astruc D Gold nanoparticles: assembly, supramolecular chemistry, quantum-size-related properties, and applications toward biology, catalysis, and nanotechnology Chem Reviews 2004 15 293 346 10.1021/cr030698+ 14719978

[B2] Ramakrishnan Murali Arumugam Karthikeyan Ramalingam Saravanan Protective effects of d-Limonene on lipid peroxidation and antioxidant enzymes in streptozotocin-induced diabetic rats Basic & Clin Pharmacol & Toxicol 2013 15 3 175 181 10.1111/bcpt.12010 22998493

